# Opportunities for microbiology citizen science: lessons learnt from three pilot projects

**DOI:** 10.1099/acmi.0.000899.v3

**Published:** 2025-04-16

**Authors:** Rachel M. Pateman, Joyce Bennett, Anthony C. Hilton, Isabella Romeo-Melody, Anton Rosenfeld, Sarah J. Routledge, Caroline Rymer, Benjamin M.C. Swift, Lucy Way, Louise Whatford, Naomi C. Wilkinson, Tony Worthington, Lewis Yandle, Ayesha S. Younis, Sarah E. West, Alan D. Goddard

**Affiliations:** 1 Stockholm Environment Institute, University of York, York, YO10 5DD, UK; 2 School of Biosciences, Aston University, Birmingham, B4 7ET, UK; 3 Garden Organic, Wolston, Coventry, CV8 3LG, UK; 4 School of Agriculture, Policy and Development, University of Reading, Reading, RG6 6AR, UK; 5 Pathobiology and Population Sciences, Royal Veterinary College, Hatfield, AL9 7TA, UK; 6 Aston Institute for Membrane Excellence, Aston University, Birmingham, B4 7ET, UK

**Keywords:** evaluation, impact, public engagement

## Abstract

Citizen science (CS) is the partnering of professional scientists and members of the public to answer real-world scientific questions. There has been huge growth in CS over the past two decades, but uptake in microbiology research has, thus far, been relatively limited. In the first part of this article, we discuss how CS is well aligned with microbiology research: sample collection methods can be simplified and used in a variety of environments; projects are expected to appeal to participants as topics are likely to be of relevance to people’s lives and interests, including the health of people and the environment; and projects can also lead to real-world impact, including the identification of new drugs or biotechnological solutions. In the second part of this article, we present our reflections on three pilot projects we have recently completed. In order for the field to grow, people need to share both their successes as well as the challenges they have faced, so that others wanting to use the method can learn from these experiences. We share simplified sampling methods for yeast strains from home brewing and baking, antimicrobial-resistant bacteria on home-grown produce and microbes on chopping boards. However, participation in our projects was limited by a range of factors, including time available and resourcing, which impacted on our ability to generate new knowledge and wider impacts. We provide recommendations for others wishing to run microbiology CS projects, including ensuring appropriate resourcing and considering the ethical implications of projects.

## Data Summary

Data for Exploring the Chopping Board Microbiome and SuperYeast are deposited within the Aston University Research Data Explorer database https://doi.org/10.17036/researchdata.aston.ac.uk.00000639.

Data for Citizen Science and Antimicrobial Resistance are deposited within the University of York Research Database: DOI: 10.15124/8fd11d9d-00e6-4e00-ba7e-cdac89cd0d3c.

The authors confirm all supporting data, code and protocols have been provided within the article or through supplementary data files.

## Introduction

Citizen science (CS) is the partnering of professional scientists with members of the public to answer real-world scientific questions. While the public have contributed to scientific endeavours for many decades [1], the term ‘citizen science’ emerged in the mid-1990s [2]. Since then, there has been an explosion of CS projects, facilitated in large part by advances in information and communication technologies [3], accompanied by an increased awareness of the benefits of the approach for science and participants and the wider socio-ecological and economic impacts it can achieve [4,5].

The majority of CS projects have taken place in the fields of environmental sciences, ecology and biodiversity [6], with increasing numbers in fields including astronomy [7] and health [8]. In contrast, there have been relatively few projects in many other fields of research. This includes microbiology, despite clear potential [9].

Our aim here is to highlight the contributions CS could make to microbial research and to provide advice to microbiologists wanting to use the approach. First, we discuss the potential microbiology CS has to generate benefits for science, participants and wider society, drawing on examples of existing projects, as well as highlighting some potential challenges to upscaling the approach. We then share our honest reflections from three pilot projects we have run and provide our recommendations so others considering using the approach can benefit from the lessons we have learnt.

## Suitability of CS for microbiology research

CS projects are known to have benefits for scientific research, the participants involved in them, and for wider society [10]. Here, we describe how these benefits can be achieved in the context of microbiology research. We draw on examples of existing microbiology CS projects we identified through searches (using the terms ‘citizen science’ AND microbiology) of the academic literature (databases Web of Knowledge and Google Scholar were searched).

### Benefits for science

A key benefit of CS is its ability to generate or process more data than would be possible by scientists working alone. The ubiquity of microbes means CS approaches are likely to appeal to microbiologists as a cost-effective way of collecting data. Data can be generated at national (e.g. tick-borne pathogens across the USA [11]) and international (e.g. in the Global Sourdough Project to characterize variations in yeast and bacteria communities [12]) scales and from a variety of environments (e.g. the diversity of microbial life on buildings in urban environments in The Microverse project; and the distribution of *Aspergillus fumigatus* fungal spores from air and soil samples in the Science Solstice and Summer Soil-stice projects [13]) to better understand and map the distribution of microbes. CS also provides exciting opportunities for innovations in sampling methods; for example, fabric socks worn over walking boots to detect Campylobacter over a wide area [14].

CS is also an excellent way to generate data from places to which scientists would not otherwise have easy access [15], such as people’s houses, gardens or even bodies. Again, this is highly relevant to much microbiology research, as evidenced by existing microbiology CS projects which have targeted sample collection in private gardens (What’s In Your Backyard? to identify new drugs in the soil) and household surfaces (Swab and Send to discover new antibiotics). CS can generate rich data by capturing information associated with samples, for example, relating to people’s perceptions or behaviours, which can be vital for interpreting results and generating recommendations. In The American Gut project, for example, over 11 000 participants submitted stool samples which were used to characterize the human gut microbiome and its relationship with health, lifestyle and diet [16].

Collaborative working between scientists and the public gives society greater ownership of research and increases understanding among scientists about issues of societal concern. In this way, CS can influence the research agenda and be a route to the democratization of science. In Good Germs, Bad Germs, for example, scientists and the public collaborated to identify and answer questions about bacteria in people’s homes [17].

### Benefits for participants

CS is particularly suited to projects where data collection methods are appropriate for public participation [18] and existing microbiology projects have shown how methods can be adapted for the collection and submission of samples from soil, air, water and a whole host of surfaces by members of the public. CS is also appropriate in situations where there is likely to be public interest in the topic that motivates participation [18]. Microbes play an important role in an array of processes that impact our lives, from nutrient cycling and climate change, to plant and animal disease and spoilage of food [19] and are likely to appeal to common citizen scientist motivations, such as wanting to help others or the environment [20].

CS projects should have benefits for both scientists and public participants [21]. Participation can lead to gains in topic-specific knowledge and skills as well as general scientific skills and understanding. Microbiology provides exciting opportunities for participants in this realm. In educational settings, in particular, participation can go beyond just sample collection. Many of the existing CS microbiology projects take place in schools, particularly in the US [22,24] and the International Microbiology Literacy Initiative has recently launched to develop a curriculum of child-centric, societally relevant microbiology teaching, including through the use of CS approaches [9].

Learning opportunities can extend beyond formal educational settings. Some projects aim to raise awareness of microbiology, including the positive role of microorganisms [22]. Others have been careful to encourage scientific thinking, for example by supporting the interrogation of personal data and the posing of new research questions in The American Gut project [16]. Bringing these learning opportunities into projects can help participants gain knowledge and decision-making skills they can use to inform related behaviours or responses [9]. Development of scientific literacy through microbiology CS [25] can also build trust in science in general or encourage people to consider science as a career [24,26].

### Benefits for society, environment and economy

Microbiology plays a role in tackling many of the key societal challenges we face and so, as with other forms of microbiology research, CS has the potential to have societal, environmental and economic outcomes beyond the purely scientific. Thus far, this potential has been most evidenced in the field of human health, where CS has contributed to the identification of new drugs [27]; understanding disease transmission to inform interventions [28,29]; and understanding issues around food safety [30,31]. Where CS can add value to traditional scientific processes is its ability to build partnerships among a variety of stakeholders, including citizens. In bringing diverse voices, experiences and priorities together to share knowledge and develop solutions, innovations that work for all are more likely to be developed [32]. CS fosters a transdisciplinary approach which, along with other holistic approaches such as systems and interdisciplinary research, are becoming more and more called on to tackle societal and environmental problems such as antimicrobial resistance (AMR) and climate change, so sustainable and effective solutions can be identified that would not have been apparent by siloed working [33,34].

### Potential challenges for microbiology CS

In addition to challenges that exist for CS in general [35], challenges for microbiology CS may include the need for specialist equipment. This may require participants to request equipment to be sent to them, collect a sample and send it back. This creates several potential ‘exit points’ for participants, meaning that initial interest in a project may not result in contribution of data [36]. Several existing projects ask participants to pay for equipment which will be a barrier to participation for many. In addition, samples will usually require laboratory processing and analysis. Most microbiology CS projects ask participants to collect and submit samples, while fewer engage participants in the analysis of samples and results. Only participating in sample collection and submission may be unappealing, especially if there is not a rapid feedback of results.

Although microbiological samples can be relatively stable, challenges exist with transport and storage, especially in mixed populations where one species may outcompete another during transport. Contamination of samples can also be an issue if the proper sampling technique is not carried out, or the materials provided are not sterile. Finally, there is always risk in growing unknown microbes in terms of potential pathogens, although these can be mitigated by careful choice of growth conditions and use of correct aseptic techniques. There are excellent resources available online which underscore the importance and practice of this for participants, which can be reinforced and confirmed in practical sessions [37,38].

Finally, acceptance of CS approaches in a particular field can take a long time to grow. Public participation in biological recording, for example, has been in place for well over a century [39], but there are still reservations in related scientific and policy-making circles about the acceptability of the method, and this is likely to be even more acute in fields such as microbiology, where the use of these methods is much more recent and less prevalent [40].

## Case studies

The authors recently completed three microbiology CS pilot projects. Here we provide summaries of each of these projects, detailing their aims, the methods we developed and implemented, and project results. In section 7, we discuss the overall successes and challenges of these projects, reflecting on the extent to which we achieved our original aims and providing some recommendations for others wishing to run microbiology CS projects.

### SuperYeast

#### Background and aims

SuperYeast arose from the large EU-funded ERA CoBioTech funded MEmbrane Modulation for BiopRocess enhANcEment (MeMBrane) project led by Aston University, which aimed to develop more robust strains for biotechnology by engineering improved biological membranes. Yeast was one focus of this project as more robust strains are sought to survive increasingly stressful fermentation conditions in the wine-making industry, arising from the impacts of climate change on sugar and alcohol concentrations [41]. In addition, yeast strains that are more tolerant to high sugar and ethanol concentrations may have characteristics that make them desirable for use in biotechnological applications such as the production of bioethanol [42].

Within the MeMBrane project, a wide variety of yeast strains from the project team’s existing collections were screened for tolerance [42]. However, it was recognized that even more tolerant strains could exist within the wider brewing and baking community, which presented an opportunity to crowdsource yeast strains. Initially, we attended the York Beer Festival in 2019 and spoke to brewers about our research. They responded enthusiastically and invited us to collect samples from empty barrels at the end of the festival. In 2020, a BBSRC grant was awarded to researchers at Aston University and the University of York to expand the project. This formed part of a UK Research and Innovation (UKRI) programme aimed at funding pilot projects to explore the use of CS in areas of UKRI-funded research where the method had not been previously applied.

The aims of the SuperYeast project were, therefore, to (i) develop and trial methods to crowdsource yeast strains and test them for desirable characteristics (ethanol and sugar tolerance); (ii) raise awareness with members of the public of the impacts of climate change on food and drink production and the role of microbes as green alternatives to petrochemical products; and (iii) potentially use resistant strains in biotech applications.

Our targets for sampling were brewing and baking activities, in particular (i) home-based activities (i.e. home brewing of wine and beer, sourdough starters for baking); (ii) small-scale commercial brewing (e.g. at beer festivals); and (iii) large-scale commercial brewing (e.g. sampling from beer and wine consumed at home). While we recognized that many of these samples would be existing commercial strains, they may not have been screened for ethanol and osmotic tolerance and so were worth sampling.

#### Methods

The project was advertised to potential participants through various channels to try to reach a wide audience. Our focus was primarily on the UK, with international participation encouraged. We used social media (Twitter) to advertise to the general public. Through our professional networks and the MeMBrane project website, we targeted people with a special interest in microbiology, and we commissioned an article and activity sheet for the Futurum Careers website and newsletter, which provides resources for young people interested in science careers [43]. We contacted breweries and special interest groups (e.g. through Facebook, Reddit) to recruit people who might have their own yeast cultures (e.g. home brewers and bakers).

**Fig. 1.**
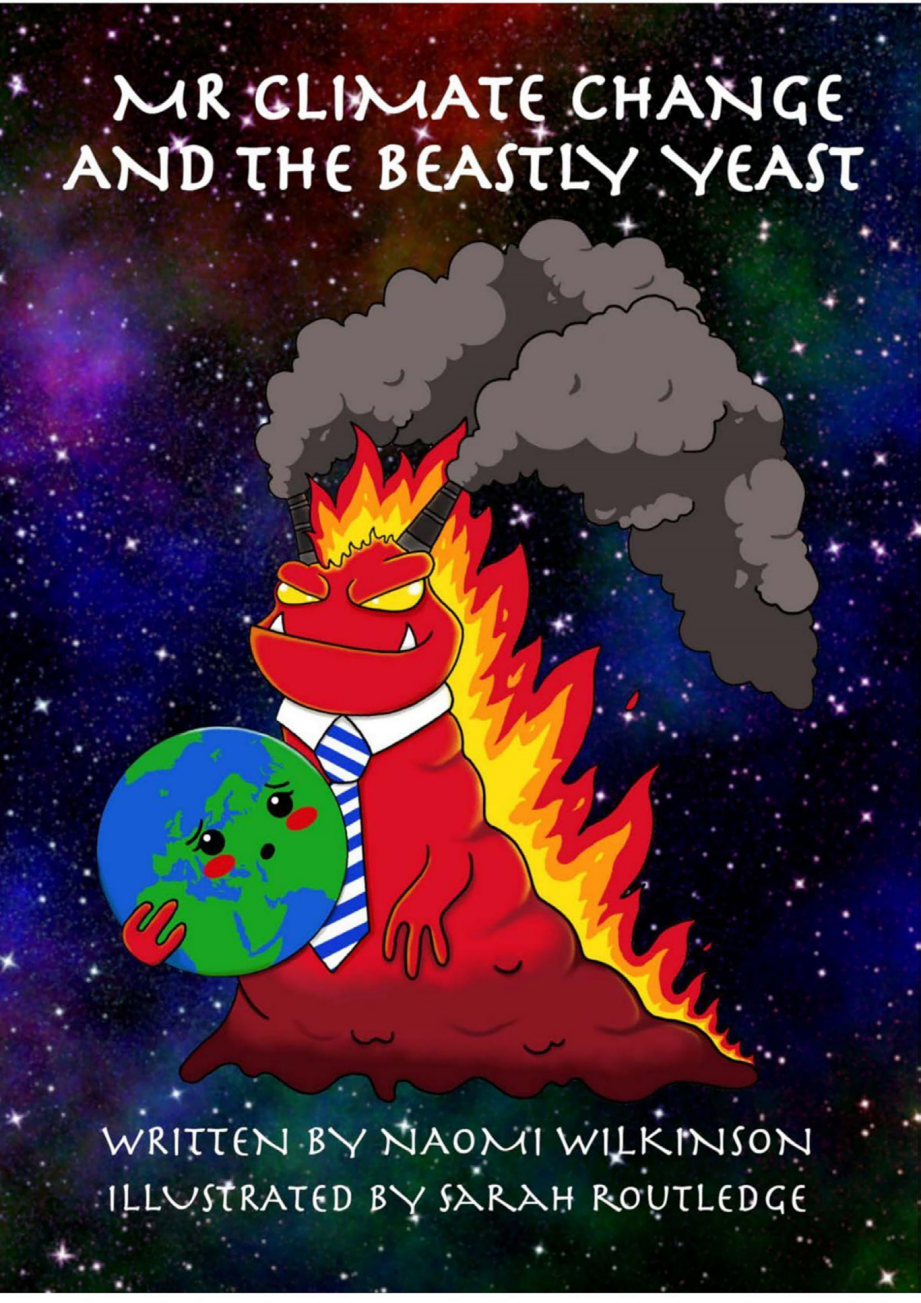
Front cover of the children’s book produced through the project. S.J. Routledge 2020.

We also targeted those who could use participation in the project as an educational activity. A lesson plan for Key Stage 3 (11–14 year olds) was developed and schools were contacted to promote this. This session involved viewing the ‘Using Biology’ music video developed as part of the MeMBrane project [44], followed by experimental work to grow yeast. We also contacted youth clubs to promote the project, and as COVID-19 restrictions continued, we targeted families looking for a lockdown activity. A children’s book about the project ‘Mr Climate Change and the Beastly Yeast’ was also produced to support participation in educational settings (Fig. 1). An e-version is available on the MeMBrane website, and a number of hard copies were procured for distribution to schools.

Potential participants were invited to contact the project team via email to request a sampling kit which they could use to take a sample and return to the project team for analysis. See Supplementary Materials, available in the online Supplementary Material for details of sample packs and sampling instructions for participants as well as protocols for analysing the returned samples for tolerance. A SuperYeast web page was created on the MeMBrane website explaining the aims and methods of the project and linking to a leaderboard with the results of the tolerance tests, allowing participants to see how their yeast ranked in the results table.

We developed a post-participation evaluation questionnaire which was sent via email to all those who submitted samples and provided contact information. This included a combination of Likert-scale questions and open-text responses. It was designed to evaluate how successful we were in meeting our aims of developing a method to crowdsource yeast strains and raising awareness of climate change and biotechnology. We asked about people’s experiences with the project, including whether they enjoyed it and would participate again, if they found the sampling method straightforward, any challenges they experienced and if they could suggest any improvements. We also asked if they learnt anything through taking part (about biotechnology, climate change and/or science) and if taking part changed their perceptions about science.

#### Results

**Fig. 2.**
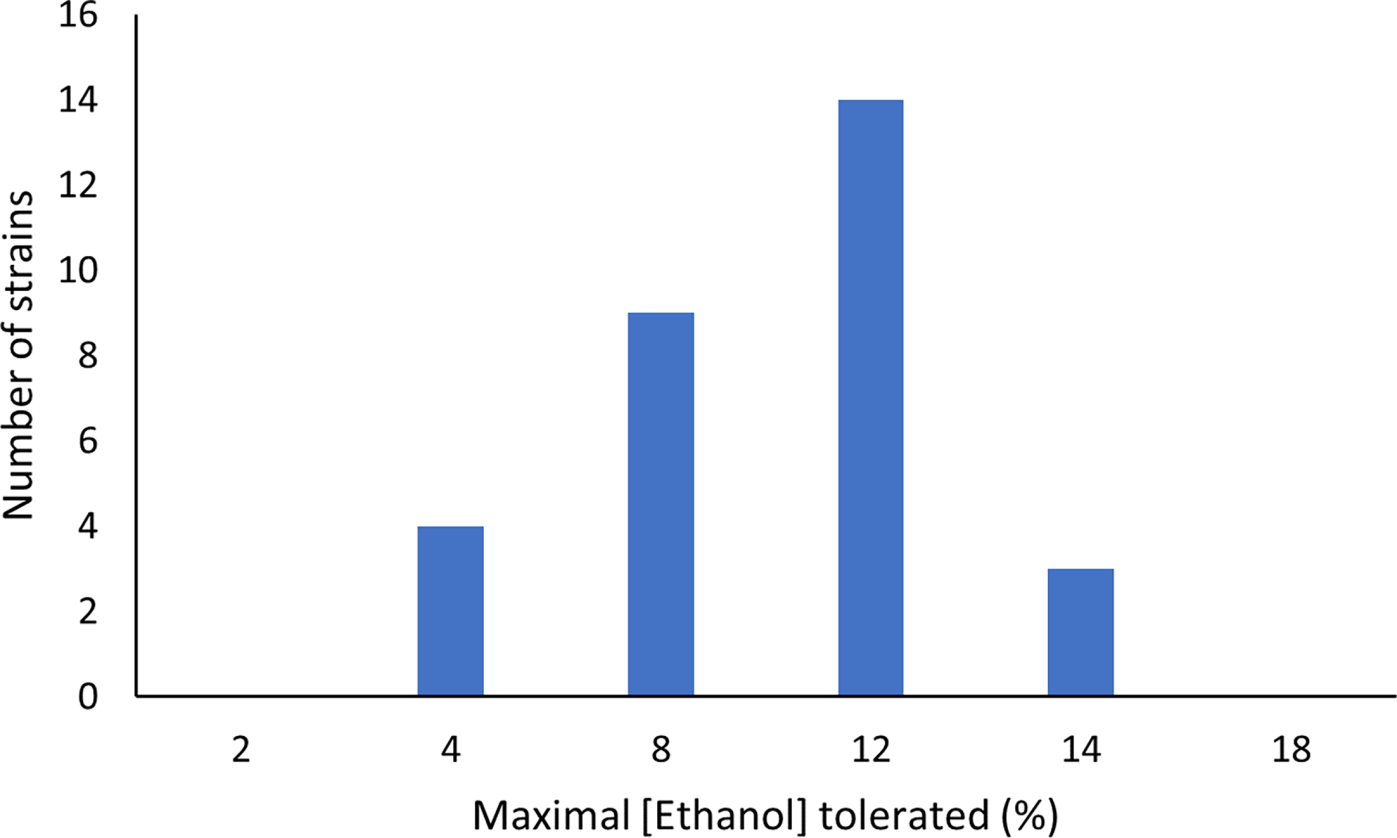
Tolerance of SuperYeast strains to ethanol. Strains were tested for visible growth on media incorporating increasing concentrations of ethanol. The concentration at which visible growth was last observed is reported. Each strain was tested in triplicate with no variation between samples observed.

Participation rates were not as high as we had hoped, largely due to the effects of restrictions enforced due to the COVID-19 pandemic. No face-to-face recruitment was possible, for example at beer festivals, which had proved successful in the run-up to the project. This meant we were reliant on remote recruitment, which proved more challenging. We received interest from secondary school teachers both in the UK and abroad. A school teacher in Australia took part with her class, sending 19 samples. A school teacher in the UK took the time to review a lesson plan and prepare for her class to take part. However, the UK lockdown prevented this from going ahead or rolling out the lesson to other schools as planned. Ten individuals submitted samples, mostly from sourdough starters or home brewing yeast. Finally, an academic researcher at the Sainsbury Laboratory in Cambridge, UK, sent 27 samples from his collection. In total, we received 52 samples, 35 of which grew. While we ranked the strains, none were as highly tolerant as the best-performing strains within the academic culture collections used for the MeMBrane project (Fig. 2). However, the strains are currently under further analysis in an ongoing project at Aston University examining the stress tolerance of yeast. All approaches such as this must be caveated around any IP or similar usage restrictions which may be placed on the provided strains. Many strains in circulation would be from industrial sources. It may be more practical to use knowledge about the strains collected in the project to then develop independent resources if industrialization is the long-term aim. In our case, we were interested in the different mechanisms of ethanol tolerance we could then use to engineer new strains [42]. Learnings from the collected strains would build into our holistic knowledge of how to do this, rather than using the strains themselves.

Unfortunately, we only received three responses to the evaluation questionnaire, and so we cannot draw any conclusions from the results. While participation was lower than we had hoped due to challenges we faced arising from the pandemic, we were able to demonstrate that we developed a method to successfully crowdsource yeast strains and test these for tolerance that could be scaled up in future projects.

### Exploring the Chopping Board Microbiome

#### Background and aims

Exploring the Chopping Board Microbiome was funded through a joint programme between the Food Standards Agency (FSA) and UKRI to pilot the use of CS approaches in projects related to the FSA’s research priority areas [45]. Our project, again led by Aston University with CS support from the University of York, focussed on understanding microbial contamination on chopping boards, and how this relates to chopping board type and kitchen hygiene behaviour, with a focus on demographic groups typically underrepresented in research.

Household surfaces are a well-known source of microbial contamination, with around 40% of outbreaks of foodborne infections in Europe occurring at home [46]. Many disease outbreaks are also caused by poor hygiene and cross-contamination from raw food, e.g. *Campylobacter* and *Salmonella* from poultry [47,48]. A key site of such microbial contamination is chopping boards [49]. While chopping board contamination and associated behaviours have been studied previously, most investigations are conducted under the supervision of researchers [50], which can lead to changes in participant behaviour. Furthermore, these studies risk only engaging ‘easy-to-reach’ groups, excluding communities who may have good practice, or undocumented challenges, in the area of food hygiene.

Aston University is unusual for the UK in that 68% of its students identify as belonging to minority ethnic groups [51]. This demographic is representative of the local community, and many students live in their family home during their studies, while others live in shared accommodation. Within this project, we worked with ambassadors from our student community to co-create a CS project which engaged citizen participants from two previously understudied households – minority ethnic and multi-occupancy – to sample chopping board microbes and report on associated kitchen hygiene behaviours. A co-created approach was taken to ensure that methods were appropriate for, and sensitive to, our citizen participants, as well as giving our student ambassadors an opportunity to gain experience in designing and implementing a real-world scientific research project.

Our aims were to determine the most effective method of chopping board sanitation in the context of our participant households in order to tailor kitchen hygiene recommendations, if needed. In doing so, we hoped to improve kitchen hygiene practices, if needed, of both our participants and the wider public. We also aimed to increase scientific capital in our student ambassadors.

#### Methods

The opportunity to participate in the project as a student ambassador was advertised to students in person by the Project Manager at the end of lectures and via the University’s e-platform for student information and, from there, via word of mouth between students. Students on Bioscience degree courses were targeted because they already possessed an interest in microbiology and the basic laboratory skills to design a robust collection protocol and assist and train other participants in laboratory work. When signing up, students could specify which aspects of the project they wanted to be involved in co-design of research questions and sampling and testing protocols; guiding citizen participants in sample collection; laboratory analysis of samples; data analysis; and dissemination of findings.

Students who wanted to be involved in the co-design of sampling methods were invited to a series of three workshops. In the first workshop, the Project Manager introduced the background and broad aims of the project. This was followed by a discussion of priority research questions the project should address. The Project Manager presented some initial suggestions which were then discussed and refined based on what students felt would be most appealing to and appropriate for the target communities. The two main research questions were agreed as ‘Does the type of material a chopping board is made from influence its microbiome?’ and ‘Does the use of a chopping board influence its microbiome?’.

The second workshop covered co-design of methods, including sample collection; capturing information about the chopping boards that were sampled and associated behaviours; and methods for laboratory analysis of samples. The Project Manager also wrote a ‘basic’ protocol that the students reviewed and refined, identifying areas that required more information and ensuring the protocol for sampling was easy to understand for participants. Following the discussions in these workshops, the Project Manager developed a final protocol for citizen participant engagement, sampling methods and laboratory analysis of samples.

At the third workshop, the co-created resources were discussed and approved by the student ambassadors so that the kits could be assembled ready for distribution. This represented the end of the methods co-creation and allowed a final ‘sense check’ of resources. The final sampling instructions can be seen in the Supplementary Materials.

Citizen participants undertook sampling under the guidance of student ambassadors. Student ambassadors collected sample kits from Aston University and took these to their household and/or households of family or friends. They then guided another member of the household through the data collection process before returning the samples to Aston University for analysis. With support from the Project Manager, student ambassadors analysed samples in the laboratory for the presence/absence of key foodborne disease-causing bacteria and bacteria originating from the human gut or skin. Instructions for this as provided to student ambassadors are detailed in the Supplementary Materials. Other student ambassadors supported with data entry and analysis.

We conducted evaluations with both our student ambassadors and citizen participants. Student evaluations consisted of pre- and post-participation focus groups and questionnaires. At the pre-participation focus group (which formed part of the first co-design workshop), we asked students what they were hoping to gain from participating in the project, and at the post-participation focus group, we asked students about their overall impressions of the project, including whether they felt the project achieved its aims, what worked well, and what could have gone better and why. We also asked what they had gained from taking part and if there was anything they had hoped to gain but had not. Our pre- and post-participation survey asked students demographic questions to assess if we had reached our target audience. It also asked about their levels of experience and confidence with different aspects of the scientific process and their interest in pursuing a scientific career. These questions were repeated in the post-participation survey to enable us to detect any changes. In the post-participation survey, we also asked for their reflections on what had gone well and challenges experienced in the project and what they felt they gained from participating.

For our citizen participants, we sent a post-participation evaluation questionnaire to all those who submitted samples. This included questions to assess the demographics of the people who provided samples; whether they enjoyed the project and would participate again; if they learnt anything through participating (about science, microbiology and kitchen hygiene); their perceptions of the sampling process (if it was straightforward and if they could suggest any changes); and if they changed their kitchen hygiene behaviour as a result of the project.

#### Results

We had interest from 45 student ambassadors across the College of Health and Life Sciences at Aston University, with a core from the Biosciences disciplines. Approximately 30 of these went on to actively engage with aspects of the project.

**Fig. 3.**
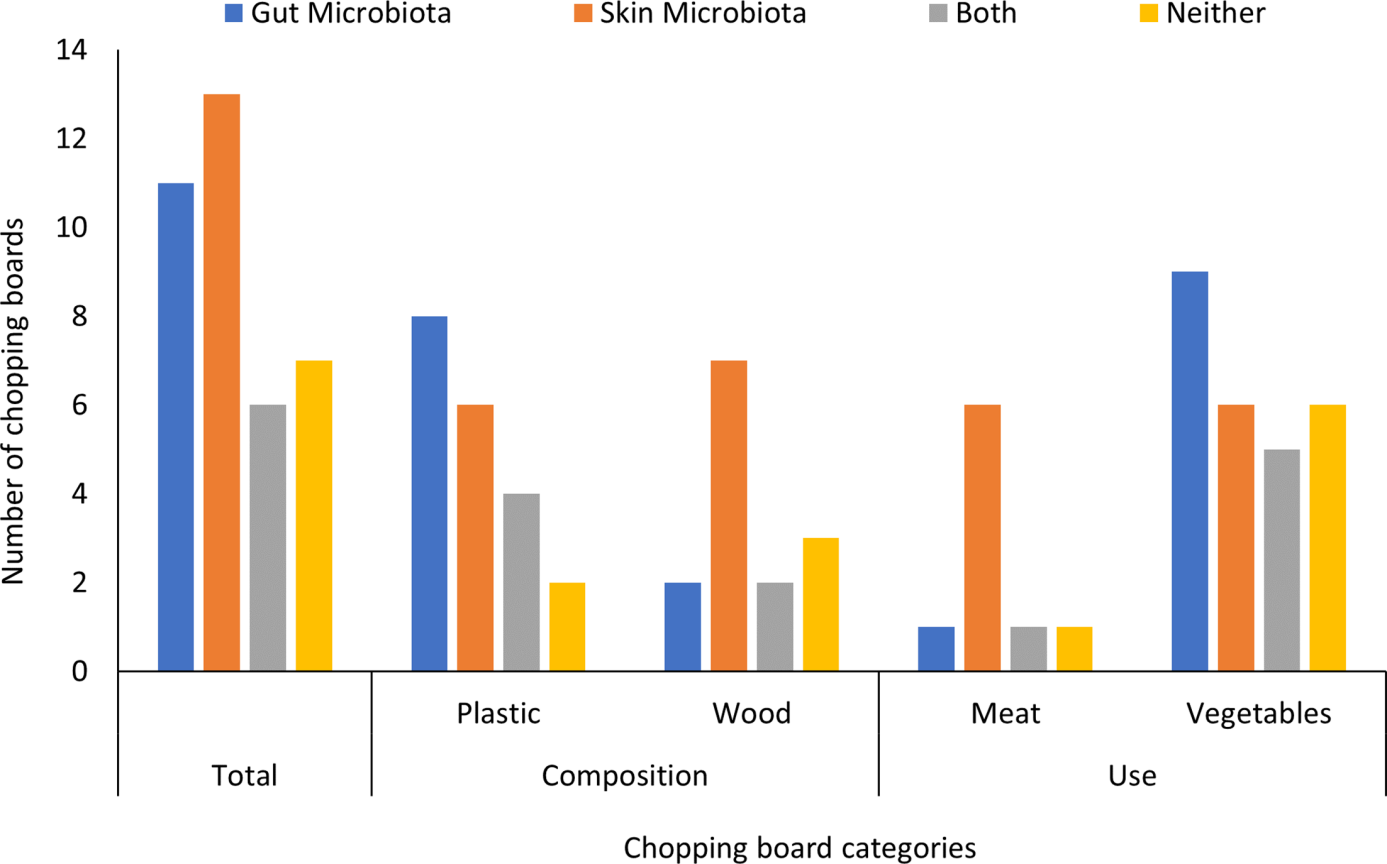
Prevalence of skin and gut microbes on chopping boards. Chopping boards were analysed for the presence of indicator microbes, and boards were designated as containing those from gut (blue), skin (orange), both (grey) or neither (yellow).

A total of 25 chopping boards were sampled to evaluate the presence of bacteria (Fig. 3). Out of all chopping boards included in this study, gut bacteria were present on 44% and skin bacteria were present on 52%. Both gut and skin bacteria were isolated from 24% of chopping boards, and 28% of chopping boards harboured neither skin nor gut bacteria. There was a significant association between chopping board material (plastic or wood) and the presence/absence of bacteria (χ^2^ = 11.151, *P* < 0.05). Gut microbiota were present on 66.67% of plastic and 20% of wooden chopping boards. Skin microbiota were present on 50% of plastic and 70% of wooden chopping boards.

Whether the chopping board was used for meat or vegetables was also significantly associated with bacterial presence/absence (χ^2^ = 13.819, *P* < 0.02). Gut microbiota were present on 14.2% of the chopping boards that were used for meat and 58% of those that were used for vegetables. This may be indicative of the care with which people clean chopping boards after meat compared to vegetable preparation. Skin microbiota were present on 85.71% of the chopping boards that were used for meat and 35.29% of those that were used for vegetables.

Unfortunately, our questionnaires did not receive high enough participation rates to produce any meaningful results. However, our final focus group with student ambassadors revealed some interesting outcomes. Students reported that they gained a better understanding of the topic than they did in lectures and that they really valued the extra practical experience they gained through the lab work. The small group working environment was particularly valued as it provided a less intimidating environment in which to ask questions and asking questions in turn built their confidence to do this more in the future. They felt the knowledge and skills they gained were transferable to other parts of their degrees and that it gave them something to put on their CV and talk about in an interview. One student reported that they were glad they had taken part in the project as the experience confirmed to them that they did not want to pursue a career in the lab, whereas for another, the experience revealed an interest in research and discovery, and they have decided to pursue a research placement and career.

### CS and AMR

#### Background and aims

This project was also funded through the FSA and UKRI pilot scheme and explored transmission pathways for AMR in the food system, focusing on home growing. There is consensus about a close association between antimicrobial use in livestock and AMR in humans [52]. Resistant bacteria can spread to humans from water used to grow produce and from cross-contamination, and also through consumption of raw fresh produce or ready-to-eat crops grown in soil amended with manure from animals whether or not treated with antimicrobials [53].

There has been a recent increase in food growing in gardens and allotments, accelerated by food security concerns during COVID-19 [54]. Unlike commercial produce, food grown at home is not regulated, so there was a desire to understand food growing practices and if these affected whether or not produce had antimicrobial-resistant bacteria on them.

The project aimed to generate evidence on the cultivation and food preparation practices of home-growers to provide further insight into the ways in which AMR moves through the food system, in particular, gaining an understanding of how people grow, and then prepare salad crops for consumption. CS is suitable for this work because it allows data collection from people’s homes about the prevalence of AMR bacteria while also providing the opportunity for participants to learn about food safety and AMR. Public knowledge about the important topic of AMR is generally low [55].

#### Methods

We took a collaborative approach to the project: participants were involved in collecting data (sending in samples) as is the case for the majority of CS projects, but in addition, they were involved in other stages of the scientific process, namely designing the research questions and methods, supporting analysis and, in some cases, disseminating findings. On the project team were Garden Organic, a charity promoting organic growing with tens of thousands of members across the UK, and growers in York. They helped shape the bid, design the research question and methods, decide on the focal crop (leafy salad crops) and remained involved throughout the project. The team also included microbiologists and CS researchers.

Growers were recruited from March to July 2022, initially by creating promotional text that could be emailed, put on posters or shared on social media, designed to appeal to different motivations for participating in environmental CS projects, particularly focusing on personal development (learning new skills), as this has been shown to be a particularly important motivation for groups typically underrepresented in CS [20]. Recognizing that time constraints are often a barrier to participation and are likely to disproportionately affect those with caring responsibilities and those in lower socio-economic groups [20], we also emphasized that participants could be involved as much, or as little, as they liked. Text was shared by Garden Organic communications channels, relevant Facebook groups, and by emailing gardening societies and community growing groups in areas with high proportions of people belonging to minority ethnic groups (based on UK census data). In order to bolster numbers further, we also shared the opportunity via the team’s internal University mailing lists, which proved a fruitful way of recruiting. In total, 124 growers were recruited.

Communication with participants took place through three core channels: a regular (approximately fortnightly) project newsletter, Question and Answer (Q and A) sessions on Zoom, and Padlet, and an online project home page which acted as a virtual noticeboard. Through the Q and A sessions and the Padlet, alongside the project meetings attended by our local grower partner and Garden Organic, the project methodology was co-designed, including swab collection methods, and an accompanying ‘swab questionnaire’ which gathered data about growing and preparation methods. See Supplementary Materials for the full protocol.

All participants had to complete a pre-project questionnaire, with the aim that they would also complete a post-project questionnaire. This included demographic information so we could assess whether we had succeeded in reaching people from groups typically under-represented in CS, information about the sites they grew in, and their knowledge of AMR.

#### Results

**Fig. 4.**
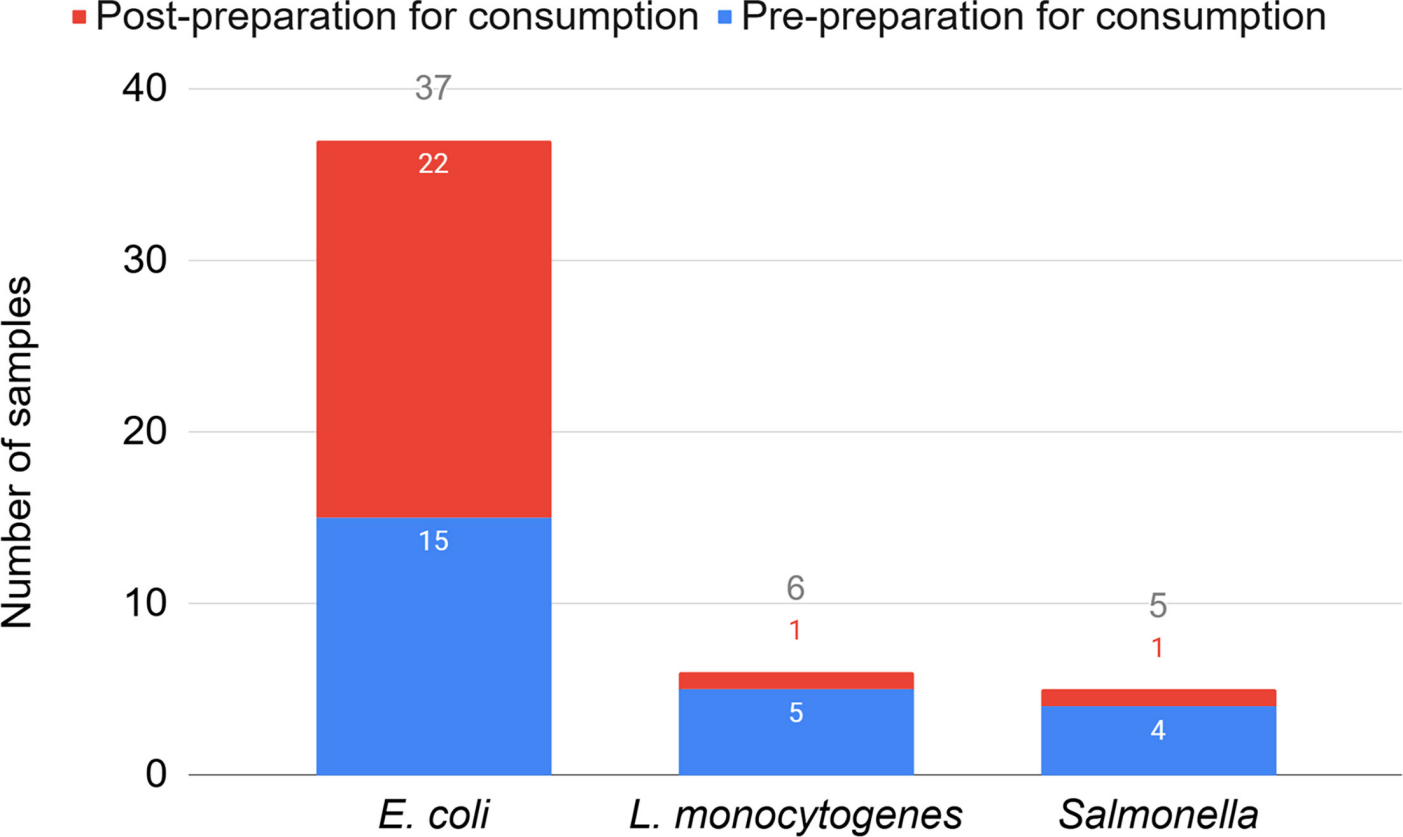
Levels of target bacteria detected and distribution across swab samples collected pre-preparation (blue) and post-preparation (red) for consumption. Note the total number of times target bacteria were detected differs from the number of samples on which target bacteria were detected because some participant samples had more than one bacterium per plate.

We had lower than hoped-for recruitment and subsequent interaction on Padlet and in our Q and A sessions, although a larger number of people (84) swabbed than we had anticipated. This yielded 127 samples pre-preparation and 127 samples post-preparation for consumption. Target bacteria (*Escherichia coli*, *Salmonella* and *Listeria monocytogenes*) were detected on 38 samples (15%), with *E. coli* found on 37 samples (15 pre-preparation, 22 post-preparation), *L. monocytogenes* on 6 samples (5 pre-preparation and 1 post-preparation) and *Salmonella* on 5 samples (4 pre-preparation, 1 post-preparation) (Fig. 4). No statistically significant relationship was found to explain the slight increase in the detection of *E. coli* in post-preparation samples, including analysis of preparation methods (e.g. washing and drying practices). In terms of antimicrobial resistance, 92% of the target bacteria were resistant to one or more of the antimicrobial agents tested, and 58% of those were resistant to three or more antimicrobials (multidrug resistance). Cefpodoxime resistance was the most common, occurring in 73% of samples which had antimicrobial resistance. We found no significant association between the presence of AMR bacteria and the use of manure, water sources or food preparation methods. There was a significant association between the potential presence of foxes, although the small sample size means this result should be treated with caution.

Unfortunately, only 33 out of 124 people completed our post-project survey, so we cannot say whether any changes in knowledge or behaviour took place. Of those who did complete the survey, there was no change in confidence relating to science, unsurprising considering 88% of those completing the post-survey had at least an undergraduate degree, and several were employed as scientists. However, 70% of post-survey respondents said they had learnt something from the project.

## Reflections and recommendations

Here, we draw together some key reflections from across our three projects. We do this by considering the extent we were able to achieve the initial aims for our projects under the outcome areas highlighted in section 5: science, participants and wider society, environment and the economy (Table 1).

**Table 1.** Intended outcomes of pilot projects

Project	Outcomes for science	Outcomes for participants	Outcomes for wider society, environment or economy
**SuperYeast**	Development of methods to crowdsource yeast strains and test them for desirable characteristics (ethanol and sugar tolerance). **[Achieved]**	Raised awareness of climate change and biotechnology. **[Partially achieved]**	Use resistant strains in biotech applications or to inform future strain engineering projects. **[Not achieved]**
**Exploring the Chopping Board Microbiome**	Generate understanding of behaviours around chopping board use by previously understudied groups (minority ethnic groups; households of multiple occupancy) and the effects of these on the chopping board microbiome. **[Partially achieved]**	A greater understanding of, interest in and skills related to science (microbiology and CS). **[Partially achieved]**Awareness and implementation of safe food hygiene practices, in particular around chopping board use. **[Partially achieved]**	Wider awareness and implementation of safe food hygiene practices, in particular around chopping board use. **[Not achieved]**
**CS and AMR**	Generation of new knowledge about practices of home growers relating to growing produce, preparation and consumption. **[Achieved]**	Increased knowledge of AMR bacteria, how it moves through the food chain, and how to modify growing or preparation practices to reduce risks. **[Partially achieved]**Increased willingness to engage with future projects and increased capacity in scientific skills, knowledge and experience. **[Partially achieved]**	Greater awareness in the growing community and other stakeholders (academics and policy makers) about how AMR bacteria move through the food system, potentially leading to improved growing or preparation practices. **[Not achieved]**Identification of future research avenues and ultimately safer growing practices. **[Partially achieved]**

### Outcomes for science

All our projects developed and demonstrated the feasibility of methods whereby members of the public independently took samples, documented associated information and returned these to scientists to analyse. These methods can be transferred to future similar projects. The involvement of experienced microbiologists to advise on project design was key to developing robust sampling methods that reduced, as far as possible, contamination of the organisms of interest. While, in SuperYeast, there was microbial contamination of some samples, this was likely due to the source of the samples, rather than the sampling method itself.

While all projects generated interesting initial findings, the depth of scientific understanding produced was limited largely due to the small number of samples returned. These were pilot projects with short time frames and limited resources focussed primarily on method development and proof-of-concept. Even accounting for this, sample sizes were disappointing. The COVID-19 pandemic proved a major disruption to the SuperYeast project; in-person recruitment was planned for based on previous positive experiences, but this had to be shifted to remote methods which proved less successful. In the Chopping Boards project, our intended sampling period was pushed back due to delays in gaining ethics approvals from an institution not familiar with CS methods. This meant that sampling unfortunately coincided with exam season for students who were our target audience, which proved a major barrier to their participation.

While there was some evidence that potential participants in the CS and AMR project were put off participating because of the topic (people were reluctant to find out what bacteria were on the produce they were growing), participation levels were higher. However, the diversity of participants was limited despite this being an aim of the project. Participation among marginalized groups is a challenge for CS more generally [56] and requires significant investment of time and resource, which was a challenge within the scope of these pilot projects. Engagement of marginalized groups in the Chopping Boards project was, however, very successful, which supports other research which has shown the benefits of engaging these groups through formal educational settings [e.g. 57]

### Outcomes for participants

While we designed robust monitoring and evaluation approaches for each project, the small numbers of participants and smaller numbers completing evaluation surveys limited our ability to assess the extent to which we achieved our intended outcomes for participants (Table 1). There were, however, participants in each study who gave supportive narrative comments. In the Chopping Board project, a focus group with student ambassadors at the end of the project showed participation promoted interest in pursuing a career in science for some, as well as benefits to academic studies (particularly additional lab time which had been limited due to the COVID-19 pandemic) and development of transferable skills. This shows the importance of using both quantitative and qualitative methods to evaluate projects.

#### Outcomes for wider society, environment and economy

Broader scale outcomes tend to require significant time and resource to achieve. In developing the initial aims for our projects, we may have been overly ambitious for what could be achieved within the scope of pilot projects. Furthermore, the challenges we faced (e.g. in terms of timing and recruitment) impacted our ability to achieve these outcomes. For example, small numbers of samples limited the scientific evidence we could generate to then make recommendations about kitchen hygiene or food growing practices. However, we did demonstrate the significant potential of what could be achieved if projects were scaled up, for example, to influence behaviour, raise awareness or to isolate novel organisms with desirable characteristics. Team members in all projects observed that CS provided engaging and effective ways to raise awareness of the issues they addressed, showing the potential for this to be achieved at a larger scale if more participants were recruited.

In order to achieve these wider impacts, it is important, where possible, for key stakeholders who can implement change to be involved in the projects. This was a particularly attractive element of the projects involving the FSA, who have since produced reports and recommendations based on the suite of CS projects they funded [58]. In this manner, impact can be generated from even short projects. Stakeholders would also include the owners of any IP from strains that could be collected from commercial sources. These are more likely to be found in food and beverage projects, but IP restrictions on use do not generally limit fundamental research, and such research can then inform future studies.

We also captured outcomes for project team members which demonstrate cascading impacts from the projects. Academics reported a desire to increase their use of public engagement and collaborative approaches to research. Student members of the project team in SuperYeast have remained in science or science communication, which they attribute to having been actively involved in the project.

### Recommendations for microbiology CS projects

Key recommendations that emerged from our projects are largely not specific to microbiology projects but CS in general, suggesting there are no unique barriers to applying CS to microbiology research and, rather, that shifts in attitudes and supporting structures are required to facilitate uptake of the method.

Our key recommendations to microbiologists wishing to use CS approaches are:

1. When seeking funding for CS projects, be aware of the significant time and resources required for the multiple stages of projects, including gaining ethical approval, (diverse) recruitment and engagement, data collection and analysis, dissemination of results and engaging with end users of data. An option is to consider writing a CS element into a larger funding proposal which might provide an opportunity to more effectively resource projects. If limited funding is available, it is important to be realistic about what can be achieved.
2. Have someone with CS expertise on the project team who can provide expertise in ethics, recruitment, retention, communication, evaluation, and so on. Ideally, also have a dedicated project officer to keep participants engaged.
3. Carefully consider how people might perceive your project and any unintended consequences. Hammes [59] describes how highlighting the presence of bacteria on showerheads and bath toys has led to headlines about ‘disgusting biohazards’. Brouwer and co-authors, however, show how careful messaging built confidence rather than concern in a CS project to test for microbial contamination in drinking water [60].
4. Consider wider ethical issues and duty of care. Make sure people know how to interpret and, if necessary, act upon the information you are giving to them. Carefully consider data ethics, in particular to whom data will be visible, especially where projects relate to potentially sensitive issues. Ensure that participants are fully briefed about health and safety, risk and COSHH and have received appropriate training for laboratory work.
5. Carefully consider intellectual property issues at the start of projects if there is a plan to use strains for commercial applications. Be transparent with participants about how samples they contribute will be used.
6. Sampling methods should be simple but robust. Trial them with a small number of participants first and, ideally, co-create methods and associated resources with participants. Publish methods as resources to guide others and share lessons learnt in the literature [e.g. 13,29, 61]
7. Be creative about how to engage people in multiple stages of a project in order to maintain their interest or extend their engagement. Consider the opportunities that might be available for involving participants in laboratory work (e.g. in ‘open days’). If this is not possible, consider other engagement opportunities such as contributing to analysing data or creating materials to disseminate findings.
8. Build a robust evaluation plan at the beginning of projects in order to capture outcomes and lessons learnt from projects. Combining qualitative and quantitative evaluation methods can add to the richness of what can be learnt. Offering multiple ways for participants to provide feedback (e.g. in person, online) can help to increase numbers of responses. Share evaluation results with researchers and practitioners to further develop CS approaches.

We also made two wider recommendations which were highly relevant to our projects. First, if CS is to be successful, it requires funding. We recommend to funders that there is a clear pipeline of funding, enabling proof-of-concept studies to be expanded with opportunities to maximize impact. We strongly encourage CS methods to be highlighted as acceptable costs within standard grants, if appropriate to the study, to encourage embedding these methods within traditional scientific research projects. Second, a significant bottleneck we experienced was gaining ethical approval from universities not familiar with CS. This was particularly challenging in co-created projects that required iterative development of resources and protocols. We recommend universities consider streamlined processes and/or seek external expertise so short projects are not unnecessarily delayed awaiting approval.

## Supplementary material

10.1099/acmi.0.000899.v3 Uncited Supplementary Material 1.

## Abbreviations

AMR: antimicrobial resistance
CS: citizen science
FSA: food standards agency
UKRI: UK research and innovation
