# Supplementary material for: Opportunities for microbiology citizen science: lessons learnt from three pilot projects
Source: Access Microbiol. 2025 Apr 16;7(4):000899.v3. doi: 10.1099/acmi.0.000899.v3 (PMC12003925; doi:10.1099/acmi.0.000899.v3)
Supplement: Uncited Supplementary Material 1. [file acmi-7-00899-s001.pdf]

Opportunities for Microbiology Citizen Science: Lessons Learnt from Three Pilot Projects

Supplementary Materials: sample collection and analysis protocols

1. SuperYeast

1.1 Sampling protocol and instructions

We initially intended to launch the project in March 2020 but COVID-19 restrictions prevented the project from going ahead at this time. The project restarted in September 2020, at which point we revised our intended sampling procedure to adapt to the ongoing pandemic. First, we switched from wet to dry sampling to avoid inadvertently spreading coronavirus. Second, sterile swabs were in great demand for COVID-19 testing, so we switched to a cotton bud in a sterile tube, which were readily available. As such, the final sampling kit consisted of a sterilised cotton bud, marked at one end (the handle), in a sterile, capped tube. Participants were instructed to collect yeast with the non-marked end of the cotton bud before sealing it inside the tube and returning it to Aston University for analysis. Participants used a swab to directly sample yeast, whether from packets, sourdough starters or sedimented yeast in brewed drinks. Full sampling instructions can be seen in Figure 1. These were sent out along with a participant information sheet, consent form, an application form requesting details about the yeast and a flyer with information about the MeMBrane project.

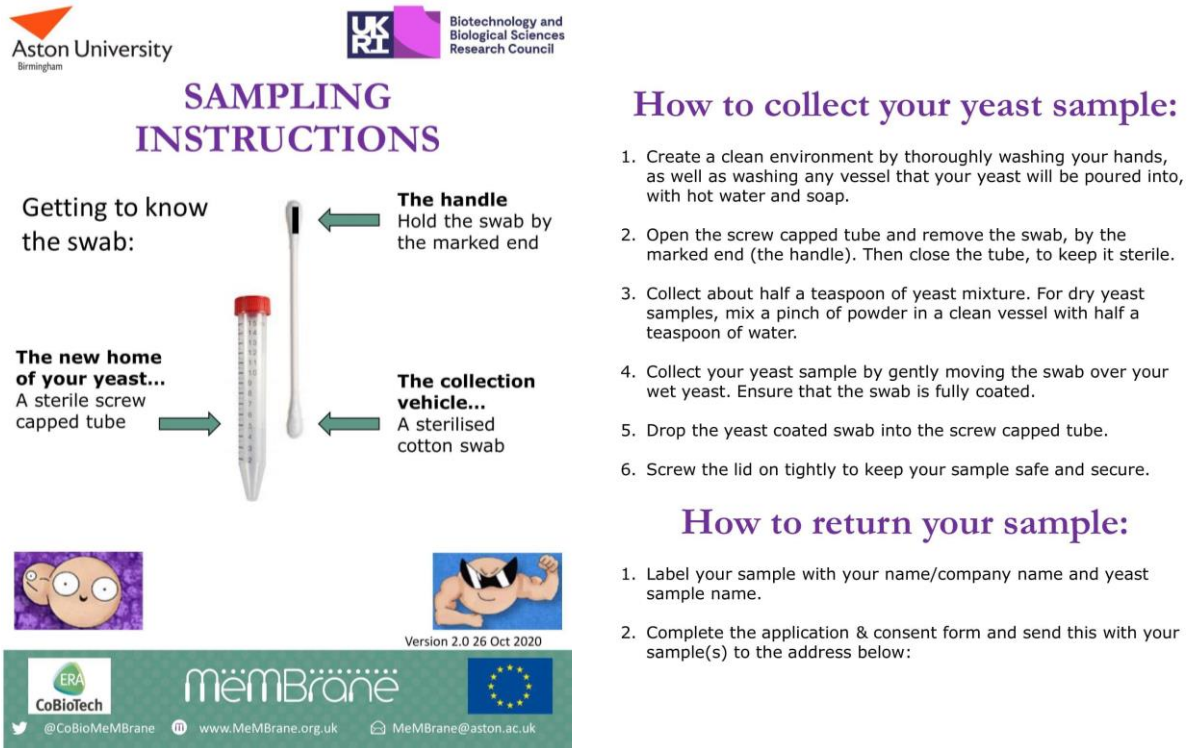

Figure 1. Sampling instructions sent to participants on request with the sampling kit.

1.2 Sample processing and data analysis

To prevent the risk of spreading COVID-19, swabs were first dried out next to a Bunsen.

1.2 1 Drop test experiments – Ethanol tolerance assay in plates

Yeast cells were grown for 24 hours in 50 mL GPY media (0.5% peptone, 0.5% yeast extract, 2% glucose) at 28°C, 180 rpm and diluted to OD600= 0.1. Then, serial dilutions of yeast cells (10-1, 10-2, 10-3) were made using GPY media. Square GPY agar (GPY media, 2% agar) plates were supplemented with varying ethanol (Fisher Chemical™) concentrations (2, 4, 8, 12, 14, 18% (v/v)), and each plate divided into three equal parts with a marker, corresponding to the serial dilutions. 10 µL of serial dilutions were pipetted onto the corresponding section of the plate, with replicates, and spread with an L-shaped spreader. The plates were wrapped in parafilm to avoid ethanol evaporation and incubated at 28°C until maximum growth was reached (up to ten days). Average area covered by colony growth in inhibitory conditions was compared to average area of growth in a positive control (absence of ethanol), to give fractional area (fa). A one-way ANOVA analysis of variance was used to check for significant differences amongst yeast species, and a Tukey test was used for comparison of means.

1.2.2 Ethanol and sucrose tolerance assay in liquid media

Yeast cells were grown for 24 hours in 50 mL GPY media at 28°C, 180 rpm and diluted to OD600= 0.1. Cells were pelleted (3,000 g, 5 minutes), washed twice with synthetic minimal media (0.68% YNB without amino acids, 2.5% glucose), then resuspended in GPY. 50 mL GPY liquid cultures supplemented with varying ethanol or sucrose concentrations, were inoculated and incubated in a shake incubator at 28°C, 180 rpm. Ethanol concentrations of 2, 4, 8, 12, 14, 18% (v/v) and sucrose concentrations of 10, 15, 20, 25, and 30% (v/v) were used. 100 µL samples were taken from the cultures every 2 hours and added to cuvettes containing 900 µL GPY, absorbance (OD600) was recorded in a spectrometer (Pharmacia Biotech Ultrospec 2000) until death phase was reached. The area under the OD-time curve of a positive control (absence of ethanol/sucrose) was compared with the areas of the test (increasing ethanol or sucrose concentration/inhibitory conditions). Fractional area (fa) was obtained by dividing the test area by positive control area (fa = (test area) / (positive control area)). NIC (non-inhibitory concentration) and MIC (minimum inhibitory concentration) parameters were calculated to compare susceptibility and resistance, respectively, to ethanol or sucrose stress. A one-way ANOVA analysis of variance was used to check for significant differences amongst yeast species for NIC and MIC parameters. Also, a Tukey test was used for comparison of means.

2. Exploring the chopping board microbiome

2.1 Sampling protocol and instructions

The following text is a copy of the sampling instructions as provided to participants. We also created a Google form for participants to complete which allowed us to gather information about the household (family home or multioccupancy house, how many people live in the house and their diets), the chopping board that was sampled (what it was made from, what it is used for, how often it is used, what condition it is in) and associated hygiene behaviour (how often it is cleaned, how it is cleaned and dried, when and how it was last cleaned).

Sampling kit contents:

- Hydrated sponges x 5
- Swabbing templates – 5 x 5 cm sampling standard x 5
- Gloves
- Information leaflet – easy to follow instructions

Preparation of the sample

Place the chopping board on a flat surface and remove contents of sampling kit. Wear the gloves included in the sampling kit before continuing, remove sampling template and place in the centre/ main area of use of the chopping board.

Sampling the chopping board

Remove the hydrated sponge from the packaging, ensuring not to squeeze to all retention of neutralising buffer. Begin sampling by swabbing inside the template. Starting at the top left corner of the template and moving horizontally down the sampling area five times to end in the bottom right corner.

Alternate the side of the sponge to now use the other side as a fresh surface to sample the chopping board and begin vertically swab inside the template, starting at the top left of the template swabbing vertically across the sampling area five times to end at the bottom right corner.

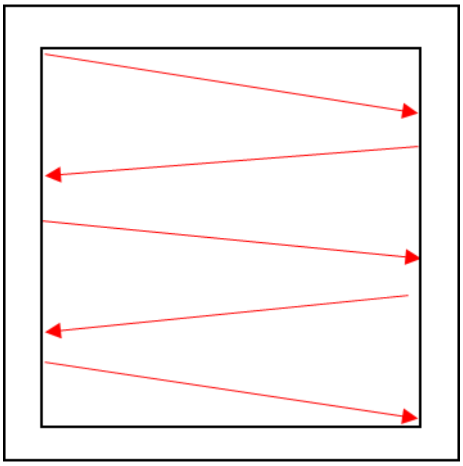

Figure 1: Diagram illustrating horizontal sampling technique.

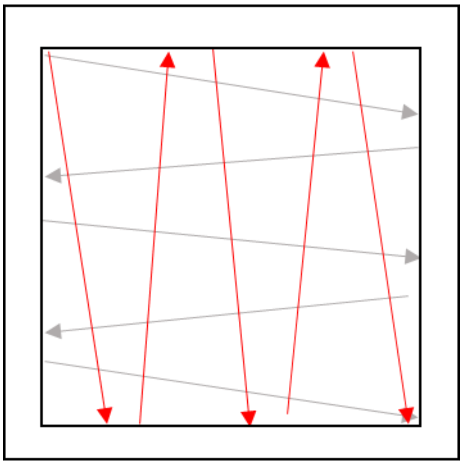

Figure 2: Diagram illustrating vertical sampling technique.

Storage and transport of the sample

Insert the swab back into the packaging and seal the bag. Store the sealed sample in the fridge until transport. Sample must be taken to the laboratory for testing within 24 hours of sample collection, to ensure maximum yield of microbiome. If participant lives nearby Aston University samples may be transferred in their own bag. Participants who require to travel longer to the University will be provided with a cool bag to transport the sample to the laboratory.

Experimental replicates of chopping board samples

Place a new sampling template on another area of the centre of the chopping board and begin sample collection with a new hydrated sponge following the sampling method. Repeat the process once more to give three samples collected from the centre of the chopping board.

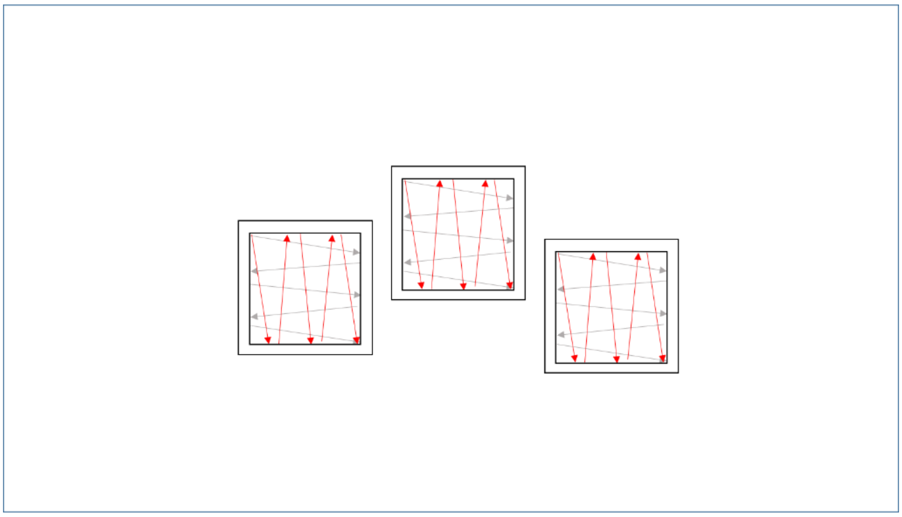

Figure 3: Diagram highlighting experimental replicates for the samples conducted on the centre of the chopping board.

Sampling the corners of the chopping board

Place new sampling template on the top left corner of the chopping board, ensuring that the sampling area reaches the edge of the chopping board. Using a new hydrated sponge begin the sampling method again.

Place the final sampling template to the bottom right corner of the chopping board, ensuring that the sampling area inside the template reaches the edge of the chopping board. Use the final hydrated sponge to sample the area.

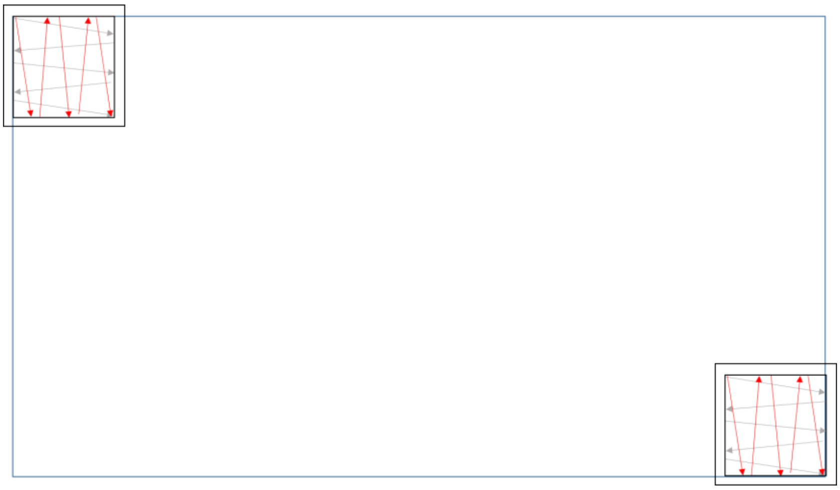

Figure 4: Diagram highlighting experimental replicates for edge samples of the chopping board.

<u>Instructions for opening and closing sample bag</u>

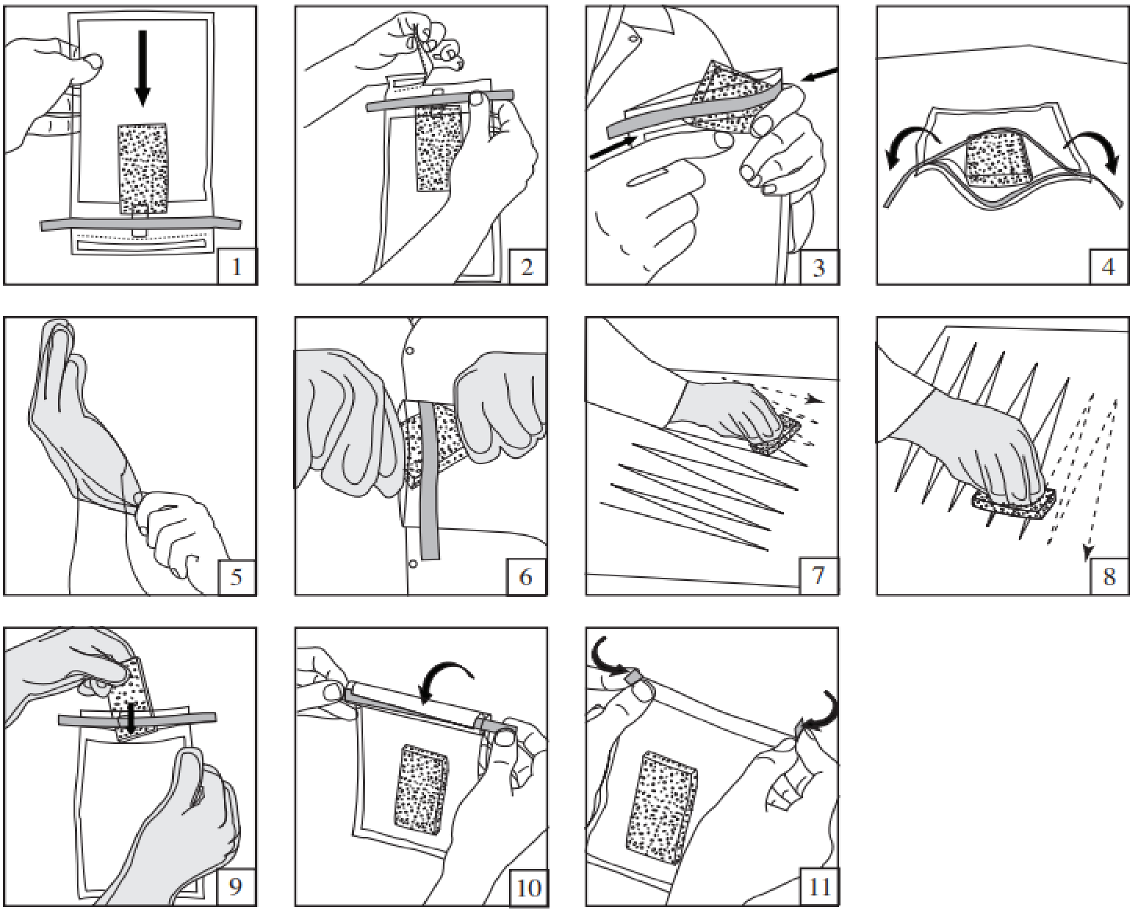

1. Shake sponge to end of bag.

2. Tear bag open.

3. Push sponge to extend from bag.

4. Bend blue wires, using red tags to form open bag.

5. Put on gloves.

6. Remove sponge.

7. Sample chopping board following the **<u>sampling protocol provided.</u>**

8. Continue to follow **<u>sampling protocol.</u>**

9. Place sponge back into the same bag.

10. Fold bag to close.

11. Fold ends of blue wires inward.

2.2 Sample processing and analysis

The following shows the instructions as provided to those running analysis of samples. This enabled us to categorise samples according to the presence or absence of skin and gut bacteria. We ran chi-squared tests to understand if there was an association between presence / absence of bacteria and chopping board type and use.

Spread plate protocol

Station checklist:

- Blood agar plates x10
- Campylobacter Blood Free CCDA agar plates x5
- Mannitol Salt agar plates x5
- Tryptose Sulfite Cycloserine agar plates x5
- Violet Red Bile Glucose agars plate x5
- Woodstick Hygiene Swabs x30
- Bunsen Burner
- Waste bin

Experimental set up:

1. Before you start ensure you are wearing your lab coat and gloves
2. Remove the sample sponges from your sample kit and number your sponges: **M1, M2, M3, E1, E2**
    a. ***This will allow us to keep the agar plates grouped correctly once they have been incubated, and ensure we keep each replicate to the correct chopping board sample***
3. Group agar plates for each sample to be tested:
    a. **Per sample sponge ensure you have: *(you should have five sets per sample kit, one for each sample sponge)***
        i. **Blood agar plates x2**
        ii. **Campylobacter Blood Free CCDA agar plate x1**
        iii. **Mannitol Salt agar plate x1**
        iv. **Tryptose Sulfite Cycloserine agar plate x1**
        v. **Violet Red Bile Glucose agar plate x1**
4. Label the agar plates
    a. **Label the back of the agar plate (*Not on the lid, if the lids are only labelled and they are mixed up/knocked over we will no longer know which sample kit the agar plate belongs to)***
        i. **Around the edge of the plate write: the date, sample kit number, sponge number e.g. 21/06/22, Kit1, M1**
            1. **Select one of the Blood agar plates and write: Anaerobic on one and Aerobic on the other**
5. Once you are ready to start, light your Bunsen burner using the electric lighter provided
    a. **Ensure the gas tap connected to the Bunsen burner is turned on**
    b. **If you do not wish to light your own Bunsen burner or are having issues lighting it, request assistance and it will be lit for you**

Streak plate protocol

6. Select one of your chopping board sample sponges and ensure you have the corresponding agar plates. While the sponge is still secure in its bag, move the sponge up to the top and squeeze the sponge to remove as much liquid as possible from the

sponge to be collected into the bottom of the bag. Carefully open the bag and keep the sponge in the bag but away from the collected liquid at the bottom of the bag

7. Insert a sterile swab into the liquid at the bottom of the sample bag and begin to transfer the sample onto a selected agar plate. Starting in the centre of the agar plate and move vertically across the left side of the agar plate, ending at the left end of the agar plate (Figure 1A)
    a. Rotate the swab to use a previously unused surface area of the swab and transfer the remaining sample on the sponge onto the right side of the agar plate, starting from the centre and vertically moving across the right side of the plate (Figure 1B)
    b. Alternate the swab and starting from the centre, horizontally move the swap up to the end at the top of the agar plate (Figure 1C)
    c. Rotate the swab once more and from the centre of the agar plate, horizontally swab down the agar and end at the bottom of the agar plate (Figure 1D)
8. Repeat spread plate technique (Figure 1) for each agar plate required for the sample sponge
9. Repeat process for each sample sponge in sample kit

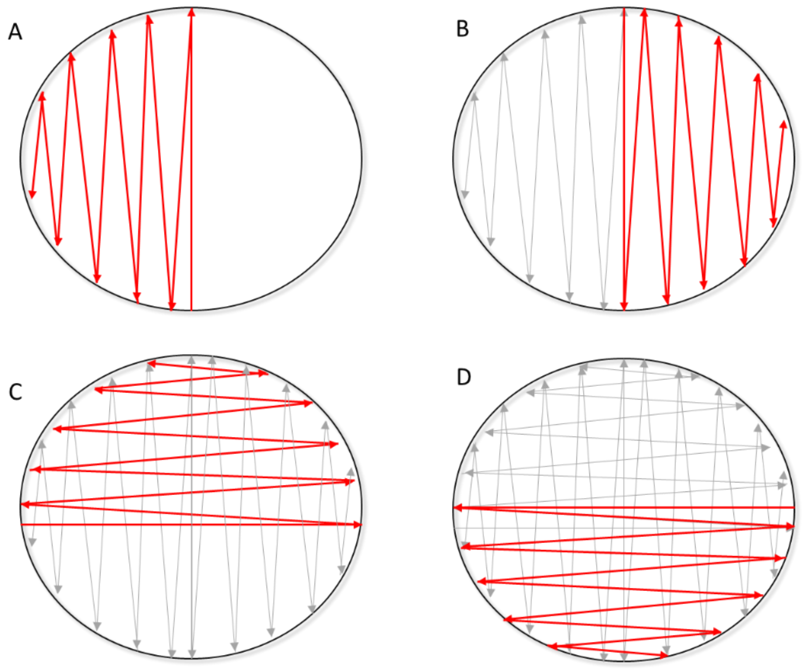

Figure 1: Diagram illustrating spread plate technique, broken down in the four main stages of using the sponge. Red lines indicate the current direction of swab movement and grey lines indicate previously swabbed area.

Storage of spread plates

Following the completion of spread plates for the sampling kits the agar plates need to be incubated.

10. Group up the blood agar plates labelled ‘anaerobic’ and the Tryptose Sulfite Cycloserine agar plates. Bring these plates up to the front of the laboratory. There you will be shown how to add a metronidazole disc onto the centre of the agar plates using tweezers.
    a. Once the discs have been added to all the plates. Tape the plates together and label the tape with the: date and sample kit number
        i. **Remember to stack the agar plates so they are upside down**
    b. These plates will be taken to be stored in an anaerobic cabinet
11. Group and tape together the following agar plates: Blood agar labelled ‘Aerobic’, Mannitol Salt agar plates and the Violet Red Bile Glucose agar plates
    a. These plates will be stores in a 37°C incubator
12. Group together the remaining Campylobacter Blood Free CCDA agar plates and bring them to the front of the laboratory
    a. Place the plates into an anaerobic jar
    b. Once the jar is full a gas pack will be added in, the jars will be sealed and stored in a 37°C incubator

3. Citizen Science and Antimicrobial Resistance

3.1 Sampling protocol and instructions

Swabs were taken once before and once after preparation for consumption. Two swabs (kept in Amies Charcoal) were labelled Sample 1 and Sample 2 and were sent out to each participant, along with a unique ID number. They were sent written instructions and a link to an explanatory video which contained the same information. They were asked to pick and swab one of the outermost leaves of the leafy salad crop, hold the stalk end and swab over the entire leaf, front and back, before placing in the tube. They were then asked to process the leaf in whatever way they usually would for consumption, and repeat the swabbing process into the second tube. They were then posted back to our team.

Below are the sampling instructions as provided to participants.

Citizen Science and Antimicrobial Resistance Swab Kit

Thank you for participating as a citizen scientist in our project!

The instructions contained in this pack relate to how to use the Citizen Science and Antimicrobial Resistance Swab Kit (enclosed) for posting to us on one of our ‘swab posting days’.

There will be two 'swab posting days', one on the 4th July and another on 11th July. Once completed, swab kits should be posted back to us on one of these swab posting days. Please be aware that we are not able to process any completed swab kits if posted after the 11th of July.

It is important that swabs are posted as soon as possible after collection. Please refer to the timeline below for guidance.

Citizen Science and Antimicrobial Resistance: Swab Timeline

Sunday 3rd July	Monday 4th July	Tuesday 5th July	Wednesday 6th July	Thursday 7th July	Friday 8th July	Saturday 9th July	Sunday 10th July	Monday 11th July
If you're posting your completed swab kit to us on the 4th, please collect your swab either today or tomorrow!	Swab posting day!						If you're posting your completed swab kit to us on the 11th, please collect your swab either today or tomorrow!	Swab posting day! **Please note that we will not be able to analyse any completed swab kits if posted after the 11th!**

Once completed swab kits should be posted as soon as possible. You can either swab and send on one of our 'swab posting days' or swab on the Sunday preceding one of our 'swab posting days' and send on the Monday.

Citizen Science and Antimicrobial Resistance is a joint project between the Stockholm Environment Institute (SEI) at the University of York, the Royal Veterinary College (RVC), the University of Reading and Garden Organic. Team members from RVC and the University of Reading will be analysing the samples you collect using this kit.

The purpose of this project is to gain an understanding of if, and how much, antimicrobial resistant bacteria are found on home-grown produce both before and after preparation for consumption. This is a pilot study, there is very little data on antimicrobial resistant bacteria on home-grown produce, which is why the project is important. We are interested in how people actually grow produce and any preparation they do before consumption, so please answer our questions honestly.

We're asking you to swab your home-grown lettuces so that we can get an understanding of what bacteria may be present and whether or not they have any resistance to antibiotics. For our purposes lettuces include other kinds of salad leaf. The key criteria are that the leaf you swab should have a good leaf area and it should not be cooked prior to consumption.

Collecting swabs using this kit is voluntary. If you choose not to complete your kit you will not be required to give a reason and there will be no repercussions.

The results from your swab samples will be stored securely and access restricted to members of the project team. Only project team members from the Stockholm Environment Institute (SEI) at the University of York will be able to identify you using your participant ID number.

Anonymised and summarised findings will be shared in a report written for the Food Standards Agency (FSA) and other outputs including scientific publications which will be made publicly available. You will not be identified in any such outputs unless you specifically write to us to ask us to identify you or your contribution.

Swabbing will take you about 15 minutes, including time to set up and to collect your sample. You will need to allow some additional time to get your swab kit to a post box and a further 15 minutes to complete our swab questionnaire.

A video demonstration of how to collect your swabs is available by scanning this QR code:

We have also put together a video to more fully explain our study which we followed with a Q and A session. Recordings of both are available on our Padlet project home now and can be accessed here [link removed post-project].

If you have any questions about these swab instructions please contact our project coordinator using [email removed]. If you're having any trouble using the QR codes or accessing the links referred to in this kit, a copy of these instructions is available on our Padlet project home which can be accessed using this link [link removed post-project].

Your swab guide
Please read through the instructions enclosed carefully.

You may like to complete step 2 (checking your kit) in advance of your chosen swab posting day so that you can ensure you've got everything.

1.

A general reminder

Citizen Science and Antimicrobial Resistance is a pilot study to gain an understanding of if or how much antimicrobial resistant bacteria are found on home-grown lettuces both before and after preparation for consumption. We are interested in gathering data which reflects the reality of home-growing so **please don't treat or prepare your lettuces any differently from how you would normally.**

Please post your completed kit on either Monday 4th or Monday 11th of July. You could swab on the Monday or the Sunday before. Please be aware that we are not able to process any completed swab kits if posted after the 11th of July.

2.

Before you get started

Before you get started please check the contents of your swab kit. In your package you should have received:

- 2 swabs (contained in tubes)
- 2 labels for your swabs (these labels will have your participant ID number and the numbers 1 and 2)
- An envelope with a prepaid return label
- This information pack

We will share the results of the completed swab kits we receive in a Zoom Q and A session as soon as we are able. As all results will be associated with an ID number we encourage you to make note of yours so that you can see the results of the sample you submit.

We've left a space here so that you can write down your participant ID number if it is helpful.

3.

Collecting your swab samples

We would like you to carefully follow these instructions to swab your lettuces and package them and send them back to our labs. Please follow the written instructions below or scan this QR code with your phone's camera to access a video showing these instructions step-by-step.

Swabbing your lettuce

1. **Label your swab tubes using the stickers provided**
2. Pick a leaf from the outside of your chosen lettuce (we recommend a lettuce but if not possible any leafy green will do).
3. Remove the swab from the tube labelled *Sample 1* and swab the entire surface of the leaf front and back

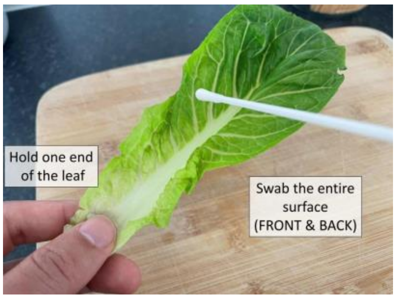

4. Replace the swab into the tube ensuring the swab touches the gel inside (this is a preservative that will keep any bacteria alive during transport and is non-toxic). Set this tube to one side - this swab is complete.
5. Now prepare your leaf as you would normally do to prepare it for eating. We don't want you to do anything other than what you'd normally do. If you don't normally do anything to your lettuce before you eat it, that is fine too!

> *We have left some space here so that you can write down how you have prepared your leaf for your second sample (this should be how you normally prepare lettuce leaves before you eat them). You will be asked to describe this in the accompanying swab questionnaire on Google Forms.*

6. Once prepared remove the swab from the tube labelled *Sample 2*
7. Repeat the swabbing over the entire surface of both sides of the leaf

	8. Replace the swab into the tube ensuring the swab touches the gel. Both of your swabs are now ready to be sent off for analysis.
**4.**	**Packaging and sending**<br><br>1. Once you have completed both swabs place them into the prepaid envelope provided and seal it<br>2. Please post your completed samples back to us on either Monday the 4th July or Monday the 11th July
**5.**	**Complete the accompanying swab questionnaire**<br><br>As soon as possible after taking your swab sample please complete the accompanying questionnaire. To access the questionnaire please scan this QR code or head to [link removed post project]<br><br>[qr removed]<br><br>If you have any trouble accessing the questionnaire please email [email removed]<br><br>If you are completing two of our swab kits please make sure that you provide us with information about each kit. This may mean that you have to submit our questionnaire twice.
**6.**	**Your results**<br><br>We anticipate that it will take approximately 2 weeks to grow the bacteria we collect from completed swab kits and analyse them for resistance. We will share the results with you at summary level in a Zoom Q and A session as soon as we are able. As all results will be associated with an ID number we encourage you to make note of yours so that you can see the results of the sample you submit.<br><br>There is a level of risk associated with the consumption of all fresh produce, and there are certain bacteria which, if present on food in certain numbers, would be of concern. We do not anticipate that the threshold levels required for this will be met but in the event that they are we will contact you to follow up using the email address we collected using the swab registration form and will liaise with the Food Standards Agency for further advice if necessary.

3.2 Sample processing and data analysis

Samples were processed within 72 hours of receipt. This involved placing into buffered peptone water, incubating at 37°C overnight, and then culturing for *Salmonella* spp according to ISO 6579-1:2017. *Listeria monocytogenes* was cultured according to ISO 11290-1:2017. *E. coli* was also cultured, with presumptive coliforms incubated at 37°C for 20 hours. Cultured bacteria were tested for antibiotic resistance using the disc diffusion method (EUCAST), with discs impregnated with apramycin (15 µg), imipenem (10 µg), cefpodoxime (10 µg), tetracycline (30 µg), trimethoprim (5 µg) and ampicillin (10 µg).

Presence / absence of the three target bacteria, and occurrence / absence of antibiotic resistance data were combined with data from the swab questionnaire, which contained quantitative and free text comments about growing methods (e.g. manure and other fertiliser use, watering regime, soil type) and preparation methods (e.g. washed, spun, dried, wiped off dirt). Analysis took place using Excel and SPSS and included Chi-squared tests to look at differences between observed and expected values.
